# A narrative review on cervical artery dissection-related cranial nerve palsies

**DOI:** 10.3389/fneur.2024.1364218

**Published:** 2024-04-18

**Authors:** Benjamin Dejakum, Stefan Kiechl, Michael Knoflach, Lukas Mayer-Suess

**Affiliations:** ^1^Department of Neurology, Medical University of Innsbruck, Innsbruck, Austria; ^2^VASCage – Research Centre on Clinical Stroke Research, Innsbruck, Austria

**Keywords:** cranial nerve palsy, vertebral artery dissection, internal carotid artery dissection, cervical artery dissection, intramural hematoma

## Abstract

**Introduction:**

This study aimed to emphasize the importance of cranial nerve (CN) palsies in spontaneous cervical artery dissection (sCeAD).

**Methods:**

A search term-based literature review was conducted on “cervical artery dissection” and “cranial nerve palsy.” English and German articles published until October 2023 were considered.

**Results:**

Cranial nerve (CN) palsy in sCeAD is evident in approximately 10% of cases. In the literature, isolated palsies of CN II, III, VII, IX, X, and XII have been reported, while CN XI palsy only occurs in combination with other lower cranial nerve palsies. Dissection type and mural hematoma localization are specific to affected CN as CN palsies of II or III are solely evident in those with steno-occlusive vessel pathologies located at more proximal segments of ICA, while those with CN palsies of IX, X, XI, and XII occur in expansive sCeAD at more distal segments. This dichotomization emphasizes the hypothesis of a different pathomechanism in CN palsy associated with sCeAD, one being hypoperfusion or microembolism (CN II, III, and VII) and the other being a local mass effect on surrounding tissue (CN IX, X, XI, and XII). Clinically, the distinction between peripheral palsies and those caused by brainstem infarction is difficult. This differentiation is key, as, according to the reviewed cases, peripheral cranial nerve palsies in sCeAD patients mostly resolve completely over time, while those due to brainstem stroke do not, making cerebrovascular imaging appraisal essential.

**Discussion:**

It is important to consider dissections as a potential cause of peripheral CN palsies and to be aware of the appropriate diagnostic pathways. This awareness can help clinicians make an early diagnosis, offering the opportunity for primary stroke prevention.

## Introduction

Cervical artery dissection (CeAD) is defined by the evidence of a mural hematoma within the arterial wall of either the carotid or vertebral arteries and can occur spontaneously (sCeAD) or in timely association with trauma. In the general stroke population, a sCeAD is rare and attributes for 1–2% of all strokes. In young individuals (i.e., under the age of 50 years of age) however, sCeAD accounts for 10–25% of ischemic strokes, making it one of the primary causes in this age group (1–4). The clinical presentation of sCeAD varies considerably with local signs and symptoms (such as head/neck pain, Horner’s syndrome, cranial nerve palsies, and pulsatile tinnitus), typically preceding ischemic stroke. As early detection of local signs and symptoms due to sCeAD followed by early treatment offers the opportunity for primary stroke prevention, understanding the clinical spectrum of sCeAD is of utmost importance. Over the years, studies and narrative reviews have focused on the frequent local signs and symptoms such as head/neck pain and Horner’s syndrome, neglecting cranial nerve palsies attributable to sCeAD (5–7). Therefore, we aimed to put the current evidence into perspective and to give an overview of the pathomechanistic as well as clinical aspects of sCeAD-related CN palsies.

## Methods

A search term-based literature review of PubMed was conducted to identify articles investigating cranial nerve palsies due to sCeAD. Search terms were “cervical artery dissection” AND “cranial nerve palsy.” Additionally, a search with the terms “cervical artery dissection” AND “insert cranial nerve” (e.g., “facial nerve”) was performed for all 12 cranial nerves. Titles and abstracts were screened, and the full texts of potentially relevant articles were obtained for review. The inclusion criteria comprised cranial nerve palsies due to spontaneous cervical artery dissection (sCeAD). Articles concerning cranial nerve palsies due to other causes such as traumatic cervical artery dissection, stroke, cancer, surgery, or local inflammation were excluded. A review of the literature was performed by the two main authors (BD and LMS). In total, 75 search results matched the inclusion criteria for this review; 22 were duplicates of different search terms. Finally, 53 publications were considered (Supplementary Table S1).

## Epidemiology/pathophysiology

**Figure 1:**
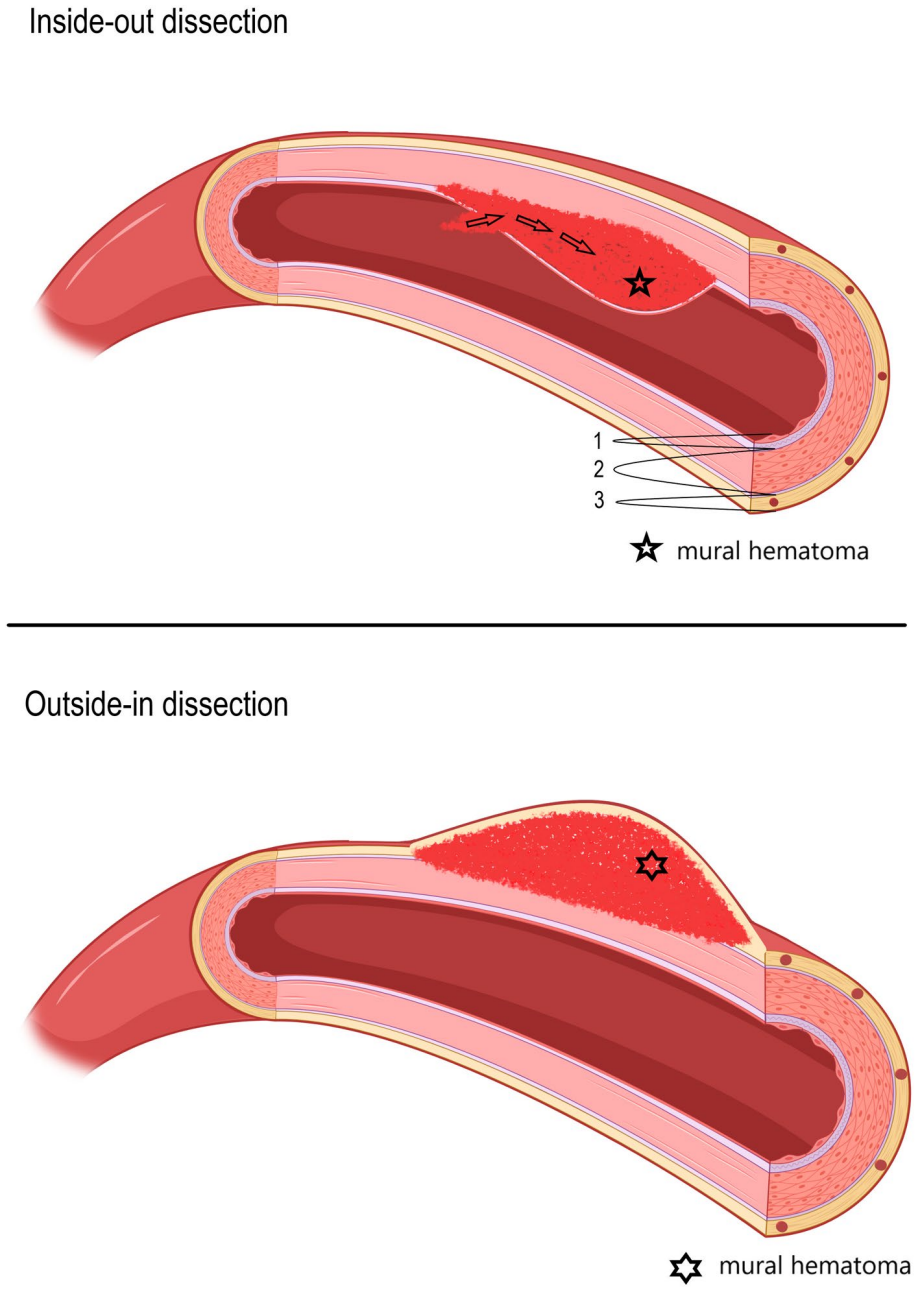
Schematic picture of two different hypothesized types of dissections (upper: inside-out; lower: outside-in), depending on the location of the mural hematoma. In the upper picture, an inside-out dissection is shown, and in the lower picture, an outside-in dissection is shown. 1—Tunica intima, 2—Tunica media, 3—Tunica externa (tunica adventitia) with vasa vasorum.

In line with the increasing availability of magnetic resonance imaging (MRI), absolute sCeAD diagnoses, especially in the vertebral arteries, have become more frequent (5, 8). The cause for sCeAD is essentially unknown. As environmental factors such as mild, non-penetrating head/neck trauma, or systemic infection are reported to be potential triggers for sCeAD, a multifactorial pathogenesis is likely (9–18). In addition, a subclinical connective tissue disorder has been proposed as a potential disease-promoting factor (19–28). Depending on the location of the pathognomonic vessel wall hematoma, two types of dissections can be differentiated: subintimal or subadventitial (29). It is assumed that subintimal dissections, which are present in over 80% of the cases, are associated with an intimal tear and subsequent anterograde blood flow from the vessel to the false lumen (inside-out theory) resulting in steno-occlusive vessel pathologies characterized by no significant diameter expansion, hence unlikely to affect nearby anatomical structures. A subadventitial wall hematoma originates from a rupture of the vasa vasorum and typically results in expansive vessel pathologies (outside-in theory) (schematic Figure 1) (4, 30–36).

## Clinical presentation—overall

The clinical presentation of patients with sCeAD ranges from asymptomatic to severe cerebral ischemic events. Hospital-based cohorts indicate a likelihood of sCeAD-related cerebral ischemia (TIA or stroke) ranging from 65 to 80%. These events usually exhibit an embolic pattern and, less frequently, occur in cases with high-grade stenosis or occlusion, including hemodynamic watershed infarcts (29, 30, 37–41). More than 80% of cases report at least one local symptom, with head/neck pain being by far the most frequent (5). The pain typically is characterized as pulling and/or dull, with mild-to-moderate progressive intensity. It often responds poorly to oral at-home analgesia and is specific to the ipsilateral side of the dissection (6). Another common local sign is the ipsilateral Horner’s syndrome, which is present in 28 to 58% of patients with internal carotid artery (ICA) dissection and can be the sole symptom in 10–12% of cases (1, 42–50). Tinnitus, often of pulsatile nature, is another possible local sign, occurring in 7–27% of cases and stems most likely from non-laminar flow in steno-occlusive sCeAD pathologies near the tympanic membrane (5, 43, 49, 51, 52).

## sCeAD-related cranial nerve palsy

Cranial nerve palsies occur in 3–12% of all patients with sCeAD and can be the sole clinical sign in 0.5% (1–4, 53, 54). Table 1 presents the clinical characteristics of published case reports with isolated CN palsies due to sCeAD.

**Table 1:** Available data of cases reporting isolated cranial nerve palsies due to sCeAD.

References	Mural hematoma localization	Dissection type	Patient symptoms	Additional LSs	Resolved (duration)
**Optic nerve (CN II)**					
Zheng et al. (55)	ICA—shortly above the bifurcation	Steno-occlusive	Loss of the inferior visual field in the right eye	None	No
**Ocular motor nerve (CN III)**					
Santos et al. (56)	ICA—shortly above the bifurcation	Steno-occlusive	Diplopia	None	Yes (1Y)
Nizam et al. (57)	ICA—shortly above the bifurcation	Steno-occlusive	Diplopia and right-sided hemiparesis	N/A	N/A
Campos et al. (58)	ICA—2 cm above bifurcation	Steno-occlusive	diplopia and right blurred vision sensation	Head/neck pain	Yes (3 M)
Wessels et al. (59)	ICA—shortly above the bifurcation	Steno-occlusive	Diplopia and left-sided hemiparesis	Head/neck pain	N/A
Hegde et al. (60)	ICA—petrous segment	Steno-occlusive	Diplopia and ptosis	Head/neck pain	Yes (2 M)
Schievink et al. (61)	ICA—2 cm above the bifurcation	Steno-occlusive	Diplopia	Head/neck pain	Yes (1 W)
**Facial nerve (CN VII)**					
Majeed et al. (62)	ICA—distal cervical segments	Expansive	Peripheral facial palsy (subsequent dysarthrophonia and dysphagia)	Head/neck pain (subsequent X, XII palsy)	No
Panisset et al. (63)	ICA—distal cervical segments	Steno-occlusive	Peripheral facial palsy, (subsequent dysarthrophonia dysphagia, and deviation of the tongue)	Head/neck pain (subsequent IX, X, XII palsy)	Yes (6 M)
Chung et al. (64)	ICA—location N/A	Steno-occlusive	Peripheral facial palsy	Head/neck pain	No
Gout et al. (65)	Both ICA—distal cervical segments	Steno-occlusive	Bilateral peripheral facial palsy	Head/neck pain	No
McCarron et al. (66)	ICA—occlusion 1 cm above bifurcation	Steno-occlusive	Peripheral facial palsy	Head/neck pain	Yes (6 M)
**Glossopharyngeal nerve (CN IX)**					
Taillibert et al. (67)	ICA—prepetrosal portion	Expansive	Dysgeusia	Head/neck pain, Horner’s syndrome	N/A
**Vagal nerve (CN X)**					
Nakagawa et al. (68)	ICA—distal cervical segments	Expansive	Dysphagia and hoarseness	Head/neck pain	Yes (4 W)
**Hypoglossal nerve (CN XII)**					
Abukeshek et al. (69)	ICA—distal cervical segments	Expansive	Dysphagia and dysarthria	Head/neck pain	Yes (3 M)
Jurkiewicz et al. (70)	ICA—distal cervical segments	Expansive	Dysphagia	Head/neck pain	No
Cruciata et al. (71)	ICA—distal cervical segments	Expansive	Dysphagia	N/A	N/A
Schmutzhard et al. (72)	ICA—distal cervical segments	Expansive	Dysphagia	Head/neck pain	N/A
Hafkamp et al. (73)	ICA—distal cervical segments	Expansive	Dysphagia	None	N/A
Lindsay et al. (74)	ICA—distal cervical segments	Expansive	Dysphagia and dysarthria	None	Yes (2 M)
Verdalle et al. (75)	ICA—distal cervical segments	Expansive	Dysphagia	Head/neck pain	Yes (2 M)
Lieschke et al. (76)	ICA—distal cervical segments	Expansive	Dysphagia and dysarthria	Head/neck pain	N/A

LSs, local symptoms; CN, cranial nerve; ICA, internal carotid artery; N/A, not applicable/unreported; Y, year; M, month; W, week.

In summary, isolated CN II, III, VII, IX, X, and XII palsies due to sCeAD have been reported in the literature. Patients with CN II or III palsy exclusively had steno-occlusive sCeAD-related vessel pathologies, while those with isolated CN IX, X, or XII palsy primarily had expansive mural hematoma. Furthermore, the mural hematoma localization typically involved more proximal segments of the ICA in those with CN II or III palsy compared to others. Solely those with sCeAD-related VII palsies had different mural hematoma localization and dissection types. In total, 80% of cases where clinical data were available reported head/neck pain as an additional sCeAD-related local symptom.

CN palsies due to sCeAD can also be present as clinical syndromes, namely, Collet–Sicard, Villaret, or Tapia syndrome (1–5, 36, 43, 54, 63, 77). Table 2 holds clinical information on such syndromes previously described as attributable to sCeAD.

**Table 2:** Available data of articles reporting clinical syndromes of cranial nerve palsies due to sCeAD.

References	Mural hematoma localization	Dissection type	Patients symptoms	Additional LS	Resolved (duration)
**Collet–Sicard (CN IX, X, XI, XII)**					
Rees et al. (78)	ICA—distal segments	Expansive	Dysarthrophonia, dysphagia, and palate deviation	Head/neck pain	No
Ruiz et al. (79)	ICA—exact location N/A	Expansive	IX-XII paresis not further described	N/A	Yes (N/A)
Saliou et al. (80)	ICA—distal cervical segments	Expansive	Dysphagia and dysarthrophonia	Head/neck pain, facial palsy	N/A
Smith et al. (81)	ICA—distal cervical segments	Expansive	Dysphonia, dysphagia, altered sensation of taste, and atrophy of left trapezius muscle	Head/neck pain	No
Zeleňák et al. (82)	ICA—extracranial segments	Expansive	Dysphagia	Head/neck pain	Yes (2 W)
**Villaret (CN IX, X, XI, XII, and Horner’s syndrome)**					
Okpala et al. (83)	ICA—distal cervical segments	Expansive	Hoarseness, dysphagia, and tongue deviation	Head/neck pain and Horner’s syndrome	N/A
Mizutani et al. (84)	ICA—exact location N/A	N/A	IX-XII paresis not further described	Head/neck pain and Horner’s syndrome	N/A
**Tapia (CN X and XII)**					
Al-Sihan et al. (85)	VA–V2 segment	Expansive	Hoarseness and vocal cord palsy tongue deviation	Head/neck pain	N/A
Introna et al. (86)	ICA—distal cervical segments	Expansive	Dysphagia and tongue deviation	None	Yes (1 W)

LSs, local symptoms; CN, cranial nerve; ICA, internal carotid artery; VA, vertebral artery; Y, year; M, month; W, week.

Clinical outcome was better in those with isolated CN palsies than those with clinical syndromes as 75% of patients with isolated palsies had complete resolution of symptoms compared to 60% of those with either Collet–Sicard, Villaret, or Tapia syndrome. However, the considerable amount of missing data on outcomes has to be mentioned.

## Discussion

**Figure 2:**
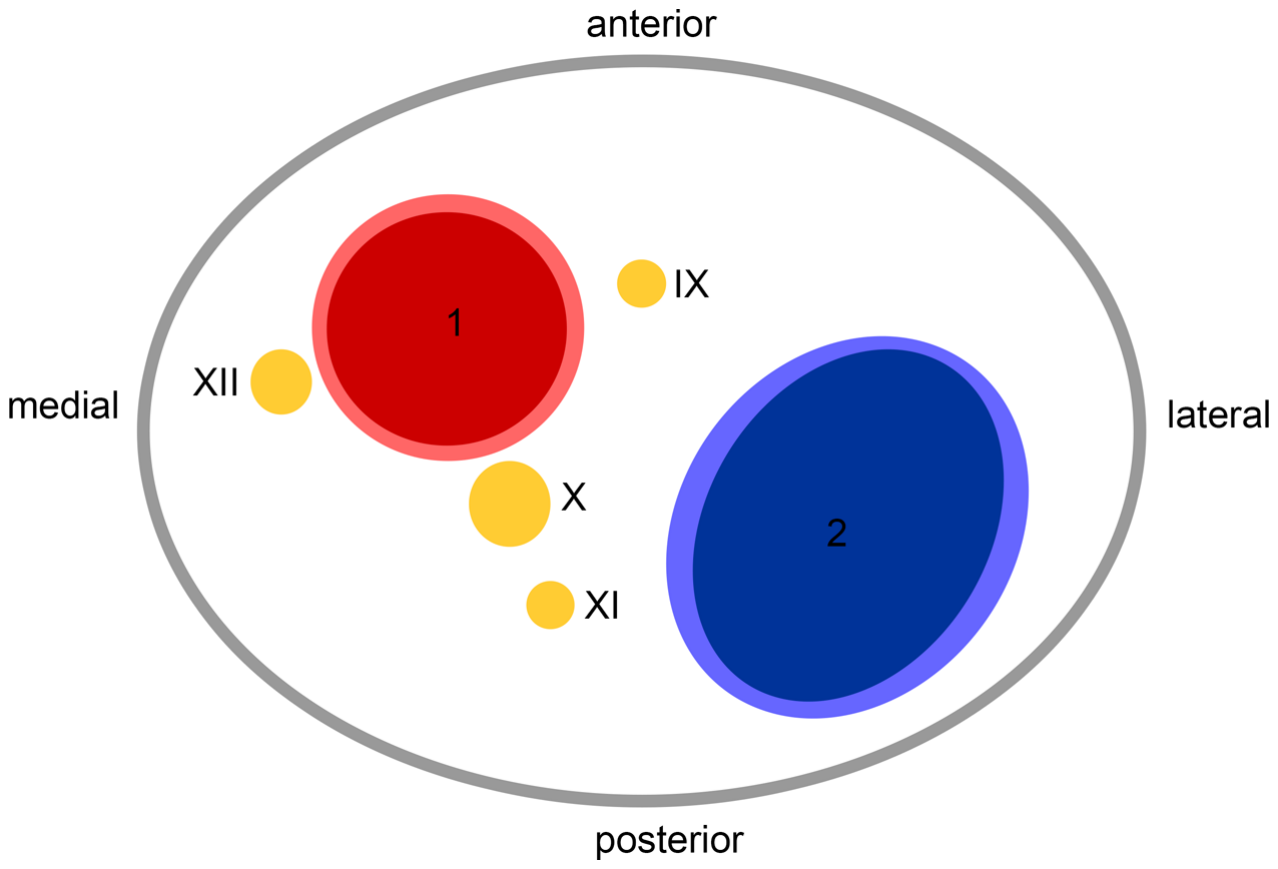
Anatomic scheme of axial view on left carotid sheath from caudal at the level of atlas; 1—internal carotid artery, 2—internal jugular vein, IX—glossopharyngeal nerve, X—vagus nerve, XI—accessory nerve, XII—hypoglossal nerve.

Hospital-based cohorts report that approximately three in four sCeAD cases suffer cerebral ischemia (1). However, local symptoms, such as head/neck pain, Horner’s syndrome, pulsatile tinnitus, and CN palsies, are the most frequent sCeAD-related symptoms and typically precede stroke (5, 47). Therefore, swift identification and management would enable primary stroke prevention. As previous studies and reviews have extensively covered more frequent local signs and symptoms in sCeAD, our review emphasizes that CN palsies are presentations that clinicians should not miss (5–7). In the literature, isolated palsies of CN II, III, VII, IX, X, and XII have been reported, while CN XI palsy only occurs in combination with other caudal CN palsies (Tables 1, 2). In view of the available literature, these palsies originate either from an expansive vessel wall hematoma causing a local mass effect on adjacent structures or as a consequence of peripheral nerve ischemia (i.e., microembolism or hypoperfusion of vasa nervorum) (36, 54, 62, 87). In cases where isolated CN palsies occur due to sCeAD, the available data depicted in Table 1 support such hypothetical pathomechanisms. CN IX, X, XI, and XII have a close anatomic vicinity to the ICA at the base of the skull and are therefore susceptible to mechanical stress (schematic Figure 2). As suggested by the published case reports in Table 1, patients who have isolated palsy of these CN also have a primarily expansive sCeAD-related vessel pathology, such as aneurysm formations (schematic Figure 1). On the other hand, those with isolated CN II or III palsy show steno-occlusive ICA pathologies due to sCeAD throughout. Therefore, the available literature supports the pathomechanism of microembolism or hypoperfusion of vasa nervorum in these cases. In addition to the solely mechanistic hypothesis of either local mass effect or hypoperfusion of vasa nervorum being causal to CN palsy, the localization of the sCeAD-related mural hematoma further supports this theory. Table 1 emphasizes that the mural hematoma in patients with CN II or III palsy involves more proximal parts of ICA, while in patients with CN IX, X, XI, or XII, the mural hematoma is primarily located at the base of the skull (schematic Figure 2). The only singular sCeAD-related CN palsy where different dissection types or mural hematoma localizations are reported is in CN VII palsy. In these cases, clinical presentation and patient history are crucial for accurate diagnosis and management.

Careful clinical examination in individuals with CN palsy can reveal additional symptoms suggestive of sCeAD. In the reported cases, 80% had additional local symptoms (i.e., head/neck pain, pulsatile tinnitus, and Horner’s syndrome), with head/neck pain being the most frequent (16 of 20). Recently, a specific type of head/neck pain associated with sCeAD (acute onset, pulling pain with mild-to-moderate intensity, which continuously increases and does not respond to oral analgesia) has been reported (6). If available, imaging should be done using MRI as sCeAD with expansive vessel pathologies may be missed by ultrasound, especially if located at more distal ICA segments at the base of the skull (i.e., where CN are adjacent—schematic Figure 2) (88). Additionally, the most important differential diagnosis—brainstem ischemia-related CN palsies—could be revealed by MRI, as this distinction is sometimes difficult to identify clinically. A key difference is that peripheral CN palsies in sCeAD mostly occur in ICA dissections, while those caused by brainstem infarction relate to vertebral artery sCeAD (5). This emphasizes the necessity of a clear diagnosis, which, given the typical localizations of sCeAD primarily in the distal segments of ICA at the base of the skull and the vertebral artery (V3), should involve T1-weighted fat-saturated axial MRI imaging, if available. Here, surrounding the vessel lumen, either an isointense (first 5 days) or hyperintense crescent-shaped rim (>5 days after onset) can be found (89). However, a reported potential false-negative rate of MRI-based infra-tentorial ischemia detection of ~10% within the first 24 h after symptom onset has to be kept in mind (90). If MRI is not available, a combination of computed tomography angiography (CTA) and ultrasound can detect other, less specific signs of sCeAD, such as long tapered stenosis, false lumen and/or intima flap, and dissecting aneurysm (1, 91, 92). This is of clinical importance, especially in counseling patients, as a small hospital-based cohort analysis has shown that brainstem stroke-related CN palsies do not resolve over time, while peripheral CN do within a follow-up of 5 months (5). This was also true for the cases discussed within this review, as 72% of patients with available clinical follow-up information showed complete resolution of symptoms over time (Table 1). Even though observational studies have shown that planned stenting of sCeAD in the subacute setting is safe, we recommend a conservative approach in accordance to current treatment recommendations due to the benign prognosis of sCeAD-related peripheral CN palsies, which is in line with current guidelines (93).

Overall, CN palsy in sCeAD is evident in approximately 10% of cases. Although their prognosis is benign, it is important to consider sCeAD and the appropriate diagnostic pathways. This awareness can guide clinicians to make an early sCeAD diagnosis, offering the chance of primary stroke prevention.

## Author contributions

BD: Writing – original draft, Writing – review & editing, Conceptualization, Methodology. MK: Conceptualization, Supervision, Writing – review & editing. SK: Supervision, Writing – review & editing. LM-S: Supervision, Writing – review & editing, Conceptualization, Methodology.

## Glossary

CeAD: Cervical artery dissection
sCeAD: Spontaneous cervical artery dissection
CN: Cranial nerve
ICAD: Internal carotid artery dissection
MRI: Magnetic resonance imaging
ICA: Internal carotid artery
VA: Vertebral artery
TIA: Transient ischemic attack
CSS: Collet–Sicard syndrome
MMA: Middle meningeal artery
APA: Ascending pharyngeal artery
ECA: External carotid artery
CT: Computed tomography
CTA: Computed tomography angiography
II: Optic nerve
III: Oculomotor nerve
V: Trigeminal nerve
VI: Abducens nerve
VII: Facial nerve
IX: Glossopharyngeal nerve
X: Vagus nerve
XI: Accessory nerve
XII: Hypoglossal nerve
PCA: Posterior cerebral artery
PCoA: Posterior communicating artery
SCA: Superior cerebellar artery
BA: Basilar artery
AICA: Anterior inferior cerebellar artery
