# Supplementary material for: A narrative review on cervical artery dissection-related cranial nerve palsies
Source: Front Neurol. 2024 Apr 18;15:1364218. doi: 10.3389/fneur.2024.1364218 (PMC11063253; doi:10.3389/fneur.2024.1364218)
Supplement: Supplementary file 1 [file Table_1.docx]

| **Supplement Table 1**: Results of our search term bases literature review (pubmed.gov). | | | |
| --- | --- | --- | --- |
| ***Search term*** | ***Total results*** | ***Excluded*** | ***Included*** |
| (cervical artery dissection) AND (cranial nerve palsy) | 182 | 129 | Total: 53  CR / CS: 46  Cohort study: 3  Review: 4 |
| (cervical artery dissection) AND (olfactory nerve) | 1 | 1 | 0 |
| (cervical artery dissection) AND (optic nerve) | 28 | 27 | Total: 1  CR / CS: 1  Cohort study: 0  Review: 0 |
| (cervical artery dissection) AND (oculomotor nerve) | 21 | 15 | Total: 6  CS / CR: 6  Cohort study: 0  Review: 0 |
| (cervical artery dissection) AND (trochlear nerve) | 5 | 5 | 0 |
| (cervical artery dissection) AND (tirgeminal nerve) | 28 | 28 | 0 |
| (cervical artery dissection) AND (abducens nerve) | 5 | 5 | 0 |
| (cervical artery dissection) AND (facial nerve) | 106 | 101 | Total: 5  CS / CR: 5  Cohort study: 0  Review: 0 |
| (cervical artery dissection) AND (vestibulocochlear nerve) | 3 | 3 | 0 |
| (cervical artery dissection) AND (glossopharyngeal nerve) | 23 | 22 | Total: 1  CS / CR: 1  Cohort study: 0  Review: 0 |
| (cervical artery dissection) AND (vagus nerve) | 99 | 98 | Total: 1  CS / CR: 1  Cohort study: 0  Review: 0 |
| (cervical artery dissection) AND (accessory nerve) | 49 | 49 | 0 |
| (cervical artery dissection) AND (hypoglossal nerve) | 90 | 82 | Total: 8  CS / CR: 8  Cohort study: 0  Review: 0 |

CS – Case series; CR – Case report
